# Development and Application of Carbon Deposition State Diagram for H-C-O Systems

**DOI:** 10.3390/ma19040648

**Published:** 2026-02-08

**Authors:** Zhimin Ding, Xiangyang Pan, Yan Zhang, Shuo Wang, Haiyan Zheng, Fengman Shen

**Affiliations:** 1 School of Metallurgy, Northeastern University, No.3-11, Wenhua Road, Heping District, Shenyang 110819, China; dingzhimin@lnist.edu.cn (Z.D.); panxiangyang12@163.com (X.P.); zhangy157@mails.neu.edu.cn (Y.Z.); 2110600@stu.neu.edu.cn (S.W.); zhenghy@smm.neu.edu.cn (H.Z.); 2 Key Laboratory for Ecological Metallurgy of Multimetallic Mineral, Ministry of Education, Northeastern University, No.3-11, Wenhua Road, Heping District, Shenyang 110819, China

**Keywords:** mass balance and chemical equilibrium diagram of H-C-O system, critical carbon deposition curve, carbon deposition state diagram for H-C-O system, carbon deposition control, specialized software for analyzing carbon deposition states

## Abstract

In both preparing and using hydrogen-rich reducing gas (H_2_RG) in direct reduction, carbon deposition occurs if operating parameters are improperly controlled, affecting the entire process. Therefore, a universally applicable method is needed to determine carbon deposition in the CH_4_-H_2_-CO-H_2_O-CO_2_ system, especially the broader H-C-O system. This study establishes a novel method based on the H-C-O system’s mass balance and chemical equilibrium diagram, alongside multi-phase/multi-reaction equilibrium principles. Critical carbon deposition point coordinates (O/C, H/C) were determined under varying conditions including temperatures typically ranging from 550 °C to 900 °C, total pressures from 0.1 to 2.0 MPa, and H_2_/CO ratios of approximately 2.0–6.9. Connecting points under identical parameters generated critical carbon deposition curves, forming a comprehensive “carbon deposition state diagram for H-C-O system”. This diagram allows precise determination of system state and carbon deposition occurrence, providing a theoretical basis for optimizing process parameters to avoid deposition. To overcome complex diagram calculations, specialized analysis software was developed. Validation using experimental and industrial data confirmed the diagram’s rationality and practicality. The diagram offers a simple, rapid, and accurate means to predict carbon deposition under specified conditions. Crucially, it guides efforts to prevent deposition while simultaneously minimizing energy consumption and costs in natural gas-based hydrogen production processes. Consequently, the “carbon deposition state diagram for H-C-O system” effectively guides actual production towards cost reduction, lower consumption, stability, and smooth operation.

## 1. Introduction

When hydrogen-rich reducing gas (H_2_RG) is produced by reforming natural gas (NG) or coke oven gas (COG), carbon deposition problems are prone to occur, leading to catalyst deactivation and reforming pipeline blockage, affecting the stable and smooth operation of production, and in severe cases, causing production stoppage. In addition, there are also carbon deposition issues in processes such as direct reduction of iron of shaft furnaces [1,2], recycling of blast furnace top gas [3,4], and hydrogen production of natural gas in the chemical industry [5,6,7,8,9,10,11].

To effectively suppress carbon deposition within the system, it is first necessary to know whether the system is in a carbon deposition state. However, to date, whether it is the carbon deposition problem in the direct reduction process of shaft furnaces [1,2] or the carbon deposition problem in hydrogen production processes of NG [12,13,14,15,16,17,18], although many scholars have conducted extensive research on carbon deposition control, most of the research has focused on the selection of process operating parameters [19,20]; the research and development of anti-carbon deposition catalysts [11], such as improving the dispersion of nickel on the catalyst surface and reducing the size of nickel grains to enhance the activity and carbon removal performance of the catalyst [13], and achieving carbon deposition control in the dry reforming reaction process by increasing the calcination temperature of Ni/MgO catalysts prepared by impregnation method [14]; and the thermodynamic calculation method for the minimum ratio of water to carbon to prevent carbon deposition [15], etc. There are few effective methods proposed for identifying whether the direct reduction process in shaft furnace or the hydrogen production process of natural gas is in a state of carbon deposition, and there is a lack of a universal method for determining whether the H-C-O system with a large quantity and wide range is in a state of carbon deposition. Although some scholars have used FactSage 7.3 thermodynamic software to analyze the thermodynamic calculations of dry reforming reaction of methane using CO_2_ and carbon deposition, proposing a residual carbon content (material fraction) of 0.01% as the critical point for carbon deposition and non-carbon deposition regions [16], the calculations are complex, and the universality is not strong.

Therefore, it is necessary to establish a widely applicable method for determining whether a carbon deposition system is present in the CH_4_-H_2_-CO-H_2_O-CO_2_ system, especially the more representative H-C-O system, for the preparation of H_2_RG in the metallurgical field, reduction process shaft furnace, blast furnace top gas recycling, and natural gas hydrogen production in the chemical field involving the CH_4_-H_2_-CO-H_2_O-CO_2_ system.

## 2. Carbon Deposition State in the H-C-O System

**Figure 1:**
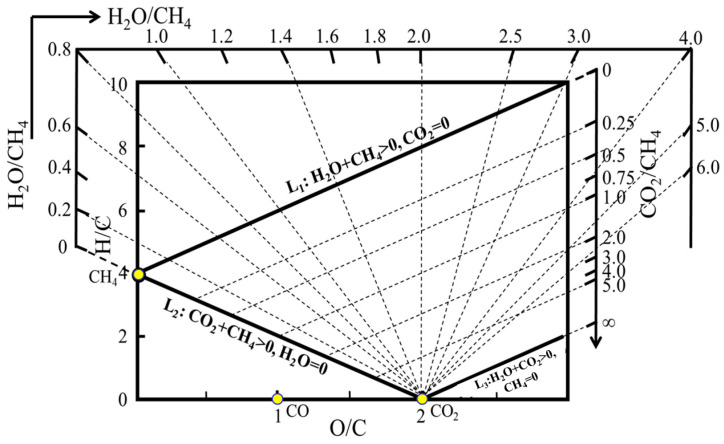
H-C-O system mass balance and chemical equilibrium diagram.

This paper determined the corresponding critical carbon deposition curves under various operating conditions, including system temperature, total pressure and H_2_/CO ratio in the system, constructed the carbon deposition state theory for H-C-O system, and developed a method to judge whether carbon deposition occurs in the gas system composed of H-C-O three elements based on “H-C-O system mass balance and chemical equilibrium diagram” [21,22,23] (Figure 1) and the equilibrium principle of a multi-reaction coexistence system, which provides a theoretical basis for selecting and controlling carbon deposition process parameters.

### 2.1. Introduction to H-C-O System Mass Balance and Chemical Equilibrium Diagram

Mass balance and thermodynamic equilibrium are used to develop the “H-C-O system mass balance and chemical equilibrium diagram” shown in Figure 1. As a first step, H_2_RG is reformulated to provide a consistent representation and to facilitate subsequent analysis. The H/C and O/C molar ratios are employed as the vertical and horizontal coordinates, respectively. Under the specified conditions, the gaseous species in the H-C-O system comprise CO (x_1_), H_2_O (x_2_), CO_2_ (x_3_), H_2_ (x_4_), and CH_4_ (x_5_). Then, the position (O/C, H/C) of the gas in the “H-C-O system mass balance and chemical equilibrium diagram” can be determined according to the equilibrium gas phase composition according to Equations (1) and (2).

(1) $$\frac{O}{C}=\frac{x_{H_{2}O}+x_{\mathrm{CO}}+2x_{CO_{2}}}{x_{CH_{4}}+x_{\mathrm{CO}}+{\;x}_{CO_{2}}}=\frac{x_{2}+x_{1}+2x_{3}}{x_{5}+{\;x}_{1}+x_{3}}=f_{1}(P_{\mathrm{tot}},T,\frac{H_{2}}{\mathrm{CO}})$$

(2) $$\frac{H}{O}=\frac{2x_{H_{2}O}+2x_{H_{2}}+4x_{CH_{4}}}{x_{CH_{4}}+x_{\mathrm{CO}}+x_{CO_{2}}}=\frac{2x_{2}+2x_{4}+4x_{5}}{x_{5}+{\;x}_{1}+x_{3}}=f_{2}(P_{\mathrm{tot}},T,\frac{H_{2}}{\mathrm{CO}})$$

**Figure 2:**
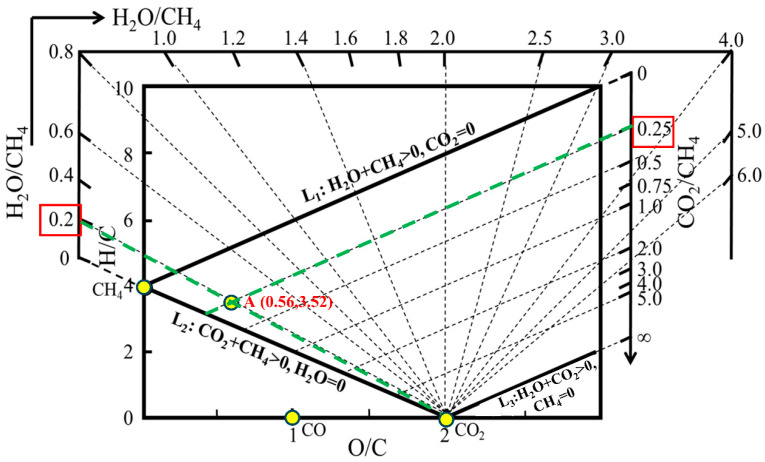
Source-gas ratio of any given-composition gas in the “H-C-O system mass balance and chemical equilibrium diagram”.

Taking CH_4_ (g) as an illustrative case, one mole of CH_4_ contains four moles of H, one mole of C, and no O. The corresponding atomic ratios are therefore H/C = 4 and O/C = 0, which place pure CH_4_ at the coordinate (0, 4). By the same definition, pure CO (g) and CO_2_ (g) are located at (1, 0) and (2, 0), respectively. To facilitate composition analysis, H_2_O/CH_4_ and CO_2_/CH_4_ molar-ratio rulers are introduced into the diagram. The H_2_O/CH_4_ ruler consists of a set of radial straight lines with constant H_2_O/CH_4_ ratios ranging from 0 to infinity, all emanating from the point (2, 0), as illustrated by the dashed radial lines in Figure 2. When the gas mixture contains only CH_4_ and H_2_O, with CH_4_ + H_2_O > 0 and CO_2_ = 0, the corresponding compositions fall along line *L*_1_ in the diagram. In an analogous manner, line *L*_2_ represents mixtures with CO_2_ + CH_4_ > 0 and H_2_O = 0, whereas line *L*_3_ corresponds to the limiting case where CO_2_/CH_4_ approaches infinity. The CO_2_/CH_4_ ruler is defined as a linear family of equal CO_2_/CH_4_ mole ratios parallel to *L*_1_ in the “H-C-O system mass balance and chemical equilibrium diagram” (parallel dashed family in Figure 1).

Based on this diagram, a graphical approach is proposed to guide the selection of reforming parameters. The method allows the location of any gas composition within the diagram, the estimation of H_2_O and CO_2_ additions required for a given H_2_RG composition starting from 1 mol of CH_4_, and the determination of source-gas ratios for H_2_RG preparation. It also provides a means to identify carbon deposition regions and to select suitable operating conditions, such as temperature and total pressure that suppress carbon formation while satisfying the requirements of the direct reduction process. As an example, pure methane (CH_4_) is used to generate a target H_2_RG (point A in Figure 2) with O/C = 0.56 and H/C = 3.52. To determine the required amounts of H_2_O and CO_2_, point A is first connected to point (2, 0), corresponding to pure CO_2_, and the line is extended backward to intersect the H_2_O/CH_4_ scale. This intersection gives an H_2_O/CH_4_ ratio of approximately 0.20. Next, a line parallel to *L*_1_ is drawn through point A, which intersects the CO_2_/CH_4_ scale at about 0.25. Accordingly, the preparation of H_2_RG at point A requires the addition of 0.20 mol H_2_O and 0.25 mol CO_2_ per mole of CH_4_. In addition, the target gas composition can also be produced using a gas mixture of NG and COG as the raw materials based on the “H-C-O system mass balance and chemical equilibrium diagram” and the lever principle (the details refer to the literature [23]).

### 2.2. Introduction to Carbon Deposition State Diagram for H-C-O System

To evaluate whether carbon deposition takes place within the system, both the critical location and the corresponding region of carbon deposition in the “H-C-O system mass balance and chemical equilibrium diagram” are identified based on thermodynamic equilibrium. The system has three degrees of freedom; therefore, the equilibrium gas composition at the critical carbon deposition state can be uniquely defined by specifying the total pressure *P*_tot_, the reforming temperature *T*, and the H_2_/CO molar ratio. On this basis, three chemical reactions are chosen as independent reactions for the equilibrium analysis.

(3) $$CO_{2}+C=2CO\;\Delta G_{2}^{o}=170,700\;-\;174.46T\;Jmol^{-1}$$

(4) $$CO+H_{2}O=H_{2}+CO_{2}\;\Delta G_{3}^{o}=\;-33,600+29.4T\;Jmol^{-1}$$

(5) $$CH_{4}=C+2H_{2}\;\Delta G_{4}^{o\;}=86,250\;-\;107.64T\;Jmol^{-1}$$

In addition, this study adopts the following set of independent equations.

(1) The H_2_/CO molar ratio in H_2_RG is defined according to the conditions of the dry reforming (DR) process:

(6) $$\frac{H_{2}}{\mathrm{CO}}=\frac{x_{4}}{x_{1}}=A$$

(2) The gas phase composition satisfies the normalization condition, with the sum of mole fractions equal to unity:

(7) $$CO+H_{2}O+CO_{2}+H_{2}+CH_{4}=\sum_{i=1}^{5}x_{i}=1$$

(3) With the total system pressure specified as a parameter:

(8) $$P_{tot}=P_{tot,\;preset}$$

where $P_{tot,preset}$ denotes the assigned total pressure of the system.

In combination with the six relations defined by Equations (3)–(8), the mol fraction of equilibrium gases in the system such as CO (*x*_1_), H_2_O (*x*_2_), CO_2_ (*x*_3_), H_2_ (*x*_4_) and CH_4_ (*x*_5_) at the critical state of carbon precipitation can be determined under the given conditions. Then, the position of the critical carbon deposition points (O/C, H/C) in the “H-C-O system mass balance and chemical equilibrium diagram” can be determined according to the equilibrium gas phase composition using the same method as shown in Equations (1) and (2) (the details refer to the literature [23]). Connecting the critical carbon deposition points with the same parameters, the critical carbon deposition curve and the carbon deposition region and non-carbon deposition region under a given condition can be obtained, that is, “carbon deposition state diagram for H-C-O system” [23,24,25,26].

**Figure 3:**
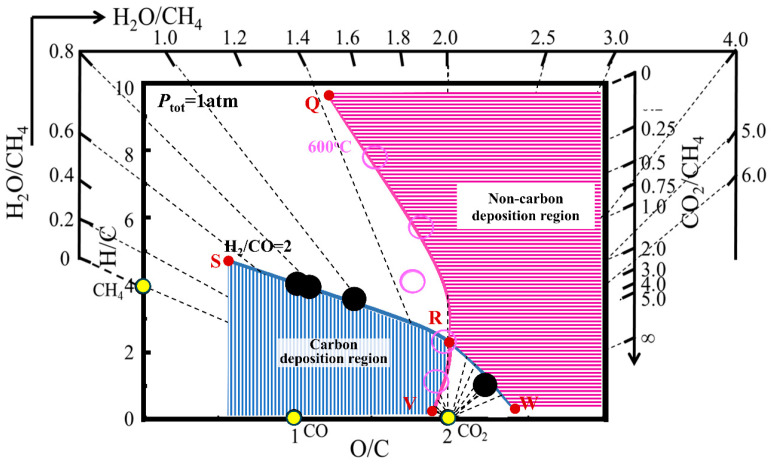
The carbon deposition state diagram for H-C-O system under the condition of 0.1 MPa, 600 °C, and H_2_/CO = 2.0.

According to the “carbon deposition state diagram for H-C-O system”, it can be determined whether carbon deposition occurs in a given production situation. For example, at a total pressure of 1 atm, a temperature of 600 °C, and an H_2_/CO ratio of 2.0, the corresponding carbon deposition state diagram of the H-C-O system is presented in Figure 3, which indicates that the horizontal region on the right corresponds to conditions without carbon deposition, whereas the vertical region in the lower left indicates the carbon deposition domain under the specified conditions. If the coordinate (O/C, H/C) of a gas phase composition is in the vertical line region, it can be considered a carbon deposition state. If the coordinate (O/C, H/C) of a gas phase composition is in the horizontal line region, it can be considered as a no-carbon-deposition state. And if the coordinate (O/C, H/C) of a gas phase composition falls in the other two regions (neither the vertical line region nor the horizontal line region), whether carbon deposition occurs is uncertain. Further thermodynamic analysis is needed for carbon deposition.

## 3. Verification of “Carbon Deposition State Diagram for H-C-O System”

### 3.1. Example I for the Prediction and Experimental Verification of Carbon Deposition Behavior of the System Under Different Process Conditions Based on “Carbon Deposition State Diagram for H-C-O System”

**Figure 4:**
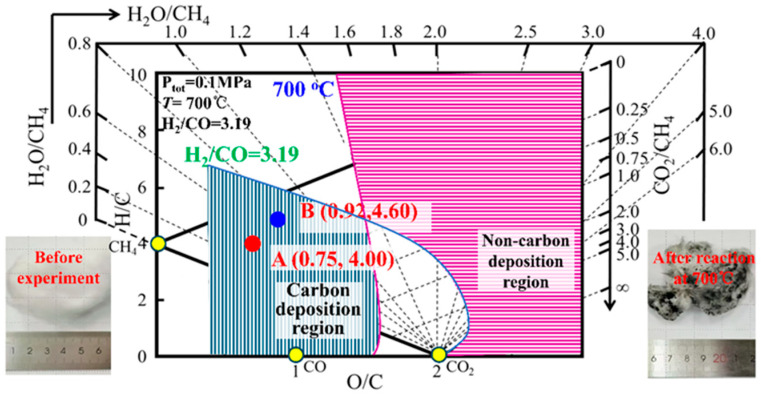
Determination of carbon deposition state in a hydrogen-rich gas atmosphere (700 °C).

To validate the predictive effectiveness of the “Carbon deposition state diagram for H-C-O system” for carbon deposition, laboratory experimental measurements [27] to determine carbon deposition in the reaction atmosphere was employed. The filling material was high-alumina media, and the experimental atmosphere used in the experiment was hydrogen-rich gas with a specific composition of 60%H_2_-30%CO-10%CH_4_. The reaction temperature was set at 700 °C, and the reaction duration was 60 min. The results obtained from the experiments are illustrated in Figure 4.

As shown in Figure 4, the initial gas composition was 60%H_2_-30%CO-10%CH_4_, with the H-C-O equilibrium point coordinates at A (0.75, 4.00). After the reaction, the gas composition became 19.95%CO-0.13%H_2_O-6.55%CO_2_-63.72%H_2_-9.64%CH_4_. Using the calculated equilibrium point of the outlet gas in the H-C-O system, the coordinates were determined as B (0.92, 4.60), and this point was in a carbon deposition region. From this, it can be concluded that under the reaction conditions of 700 °C and 0.1 MPa under the hydrogen-rich gas remains in the carbon deposition region both before and after the reaction. The aforementioned example demonstrates that the carbon deposition state theory has strong predictive capability for the carbon deposition state under a given reaction atmosphere.

### 3.2. Example II for the Prediction and Experimental Verification of Carbon Deposition Behavior of the System Under Different Process Conditions Based on “Carbon Deposition State Diagram for H-C-O System”

**Figure 5:**
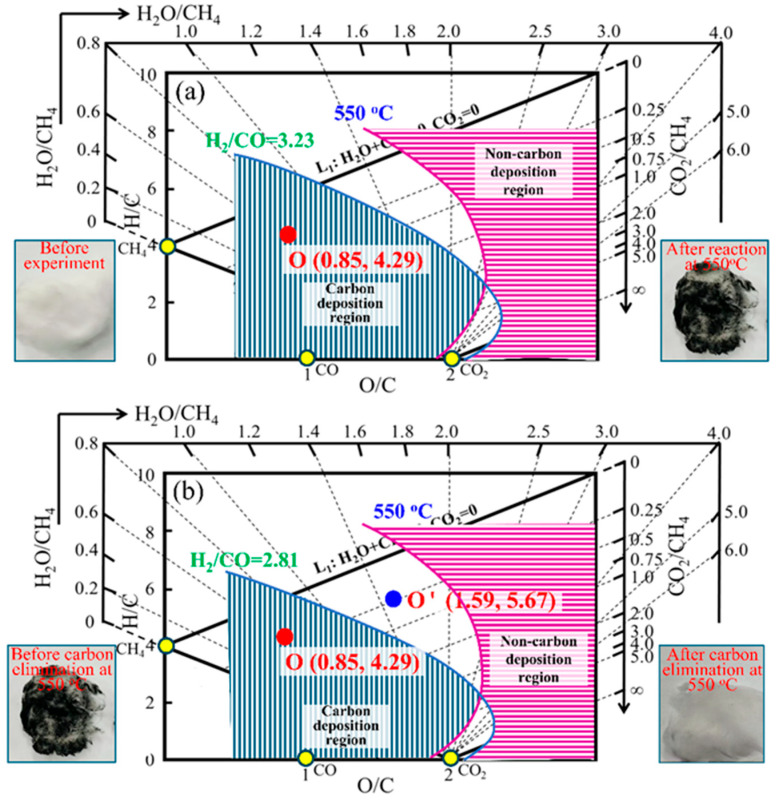
Example II for the prediction and experimental verification of carbon deposition behavior of the system under different process conditions based on “carbon deposition state diagram for H-C-O system”: (**a**) gas positions in carbon deposition state diagram and images of quartz wool before and after experiment (550 °C); (**b**) gas positions in carbon deposition state diagram and images of quartz wool before and after carbon elimination (550 °C).

Figure 5 shows the analysis of whether carbon deposition will occur or not under different process conditions and the verification of laboratory experiment using the “carbon deposition state diagram for H-C-O system”. Point O in Figure 5a is the position of the system in the “carbon deposition state diagram for H-C-O system” under the reaction conditions of 0.1 MPa, 550 °C, and gas phase composition of 58.58%H_2_-18.11%CO-8.31%CO_2_-14.78%CH_4_-0.22%H_2_O-0.00%N_2_ (H_2_/CO = 3.23). It can be judged that carbon deposition phenomenon will occur in the system; then, the verification experiment under the same conditions was carried out. The photo in Figure 5a is the photo of quartz wool filled in the reaction crucible before and after the verification experiment; it can be seen from the photo that the quartz wool changes from white before the reaction to black after the reaction, which confirms that carbon deposition indeed occurred under this condition. Furthermore, the quartz wool carbon-deposited under the above conditions was placed at 0.1 MPa, 550 °C, and the gas phase composition was 80%N_2_-20%H_2_O for carbon elimination. The outlet gas phase composition was 6.00%H_2_-2.13%CO-0.70%CO_2_-1.38%CH_4_-3.15%H_2_O-86.67%N_2_ (H_2_/CO = 2.82) and the (O/C, H/C) coordinates of the gas composition are located at point O’ in Figure 5b, and it can be seen from the figure that point O’ has left the carbon deposition region, indicating that the precipitated carbon can be eliminated under this condition. At the same, comparing the photos of quartz wool before and after carbon elimination in Figure 5b, it is confirmed that the system does have the effect of carbon elimination.

### 3.3. Example III for the Prediction and Experimental Verification of Carbon Deposition Behavior of the System During the CH_4_ Reforming Process Based on “Carbon Deposition State Diagram for H-C-O System”

Table 1 shows some experimental data on CH_4_ reforming. The first row in Table 1 is the composition of inlet mixed gas (H_2_/CO = 4.54), the temperature (800~900 °C), and pressure (1.6 MPa) of the system. The experimental results indicate that carbon deposition occurs at the inlet of the reaction tube under these conditions. To eliminate carbon deposition, the method of adding water vapor into the experiment was adopted. The results show that no carbon deposition is found at the inlet when the amount of water vapor added is three times the methane content (the gas composition after adding water vapor is listed in the second row of Table 1), indicating that adding three times the water vapor can prevent carbon deposition.

**Figure 6:**
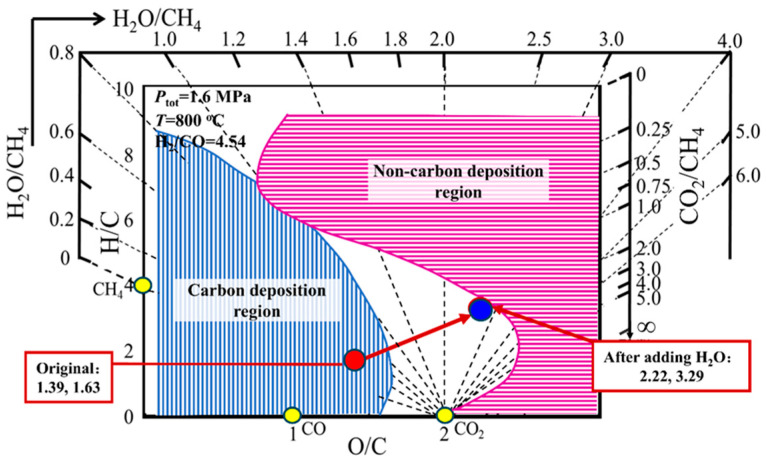
Example I for the prediction and experimental verification of carbon deposition behavior of the system during the CH_4_ reforming process based on “carbon deposition state diagram for H-C-O system”.

The carbon deposition state theory for the H-C-O system elucidates the correctness of the above results. First, the carbon deposition state diagram for H-C-O system under the conditions of 1.6 MPa, 800 °C and H_2_/CO = 4.54 was drawn and shown in Figure 6, and then coordinates (the O/C and H/C values) corresponding to the original mixed gas (Row 1 in Table 1) and the mixed gas after adding three times of water vapor (Row 2 in Table 1) respectively were calculated. From the carbon deposition state diagram for the H-C-O system, it can be seen that the original gas coordinate points (1.39, 1.63) are located in the carbon deposition region of the diagram, indicating that carbon deposition must occur in the given gas phase composition. However, the coordinate points (2.22, 3.29) are located at the edge of the non-carbon deposition region after adding three times of water vapor into the system, which indicates that carbon deposition will not occur after adding three times of water vapor into the system. Therefore, it can be seen that using the “carbon deposition state diagram for H-C-O system” can quickly and accurately determine whether carbon deposition will occur in the H-C-O system under a given condition.

These results indicate a strong consistency between the experimental observations and the outcomes derived from the H-C-O carbon deposition state diagram, confirming its soundness. Therefore, the “carbon deposition state diagram for H-C-O system” can be used to quickly and accurately determine whether carbon separation will occur for the H-C-O system under the defined conditions.

## 4. Applications of “Carbon Deposition State Diagram for H-C-O System”

### 4.1. Efforts to Reduce Energy Consumption and Cost of Reforming Unit in Hydrogen Production Process from Natural Gas

**Figure 7:**
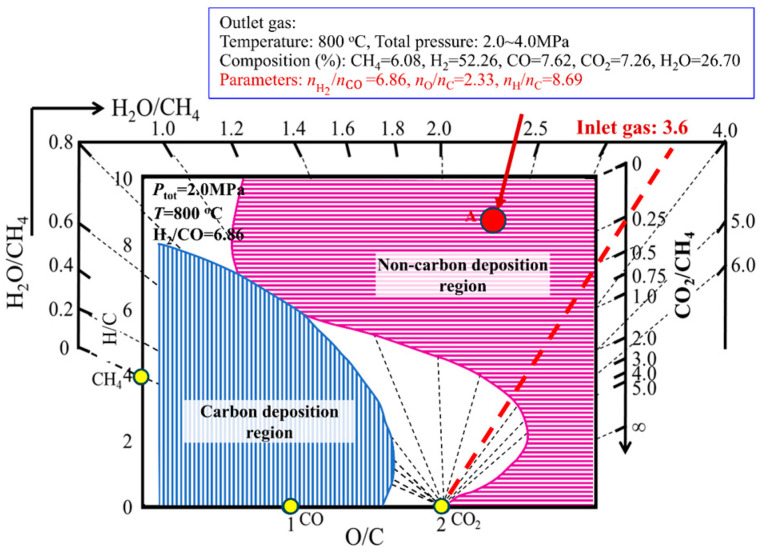
Application of the H-C-O system carbon deposition diagram to a natural gas-to-hydrogen process.

To prevent carbon deposition in the reforming unit, operation with a high water-to-carbon ratio (H_2_O/CH_4_) is generally adopted. Currently, the water-carbon ratio is approximately 3.6. Although this operation can prevent carbon deposition in the reforming unit, the corresponding consequence is that the energy consumption and cost of the reforming process are relatively high. According to the production data of hydrogen production process from natural gas, the H_2_O/CH_4_ ratio at the inlet of the reforming unit is 3.6, the outlet gas pressure is 2.0~4.0 MPa, the temperature is 800 °C, and the composition is CH_4_ = 6.08%, H_2_ = 52.26%, CO = 7.62%, CO_2_ = 7.26%, H_2_O = 26.70%. The carbon deposition state diagram for H-C-O system under the most conducive carbon deposition condition (*P*_tot_ = 2.0 MPa, *T* = 800 °C, H_2_/CO = 6.86) was drawn and shown in Figure 7. Then, the outlet gas coordinate point (2.33, 8.69) was calculated and drawn into the carbon deposition state diagram for H-C-O system (Figure 7). Since this point is in the non-carbon deposition region (horizontal line region), it is considered that no carbon deposition occurs in the reforming unit during the current hydrogen production process from natural gas. However, further analysis shows that the gas coordinate point at the outlet of the reforming unit is in the non-carbon deposition region and far away from the critical carbon deposition curve, which indicates that the water-carbon ratio of 3.6 adopted in the production process is too high, and it can be lowered appropriately, thereby reducing process energy consumption and production cost.

### 4.2. Carbon Deposition Behavior in COG-Based Shaft Furnace Direct Reduction

**Figure 8:**
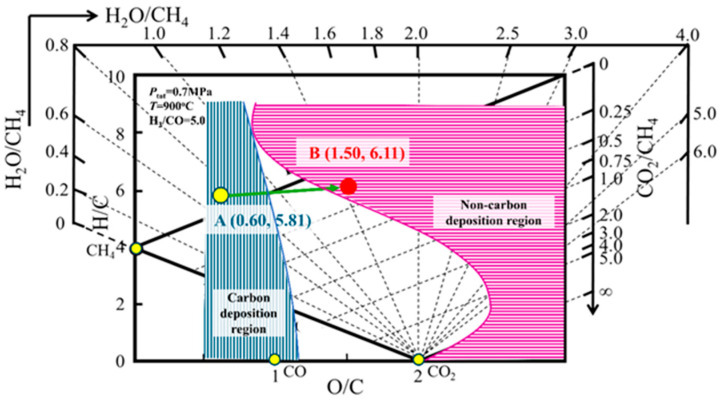
Carbon deposition states at the shaft furnace inlet and outlet derived from the “carbon deposition state diagram for H-C-O system”.

The “H-C-O system carbon deposition state distribution diagram” characterizes carbon formation behavior during COG-based shaft furnace reduction. Based on Table 2, the “H-C-O System Carbon Deposition State Calculation Special Software” [28] generated the deposition state diagram (Figure 8) at 900 °C, 0.7 MPa, and H_2_/CO = 5.0, Subsequently, the gas states at the furnace inlet and outlet were mapped using their respective (O/C, H/C) coordinate. A point (inlet gas) and B point (outlet gas) in the figure were located respectively. Since a point was located in the carbon deposition region and B point was located in the non-carbon deposition region, it is evident that carbon deposition at the inlet of the COG-based direct reduction process of the shaft furnace, while there should be no carbon deposition at the outlet. In fact, Table 2 reveals that the H_2_O content in the gas composition at the outlet is 20.4%. The higher H_2_O content inhibits carbon deposition at the outlet of the reaction tube.

## 5. Conclusions

(1) Within the H-C-O elemental framework, hydrogen-rich reducing gas compositions occupy a well-defined thermodynamic domain governed by mass balance and equilibrium constraints. This representation clarifies the position of a given gas mixture within the overall compositional space. Based on this framework, practical adjustment strategies can be formulated. These include adding oxidizing components or coordinating multiple gas sources to reach target compositions while preserving thermodynamic consistency.
(2) Carbon formation in multi-component H-C-O systems does not occur randomly. Instead, it emerges when the gas composition crosses specific boundaries in O/C–H/C space under given temperature, pressure, and H_2_/CO ratio conditions. These boundaries separate stable operating regions from deposition-prone regimes and offer a coherent thermodynamic interpretation of carbon behavior in gas mixtures typical of direct reduction and hydrogen production processes.
(3) The validity of these thermodynamic boundaries is supported by both experimental observations and industrial operating data. When implemented in a computational tool, the framework becomes straightforward to apply in practice. It helps operators recognize safe operating windows, respond to compositional fluctuations, and maintain stable operation. In this way, the proposed approach contributes to improved energy efficiency, lower operating costs, and enhanced process reliability in natural gas-based hydrogen-related systems.

## Figures and Tables

**Table 1:** Experimental data of CH_4_ reforming.

	Composition, %							Pressure, MPa	Temperature, °C	O/C, -	H/C	H_2_/CO
	H_2_	CO	CH_4_	N_2_	CO_2_	H_2_O	Content					
Mixture (Inlet)	20.22	4.45	21.58	2	51.76	0.00	100.01	1.6	800~900	1.39	1.63	4.54
Mixture (H_2_O/CH_4_ = 3)	20.22	4.45	21.58	2	51.76	64.74	164.75	1.6	800~900	2.22	3.29	4.54

**Table 2:** Inlet and outlet gas compositions of the shaft furnace reduction section.

Composition	H_2_, %	CH_4_, %	CO, %	CO_2_, %	H_2_O, %	N_2_, %	Sum, %	O/C	H/C	H_2_/CO
Inlet gas	46.8	20.2	9.2	2.4	5.1	16.2	99.9	0.60	5.81	5.09
Outlet gas	37.4	12.8	8.4	6.1	20.4	14.7	99.8	1.50	6.11	4.45

## Data Availability

The original contributions presented in this study are included in the article. Further inquiries can be directed to the corresponding author.
